# Pulmonary arterial hypertension in scleroderma: care gaps in screening

**DOI:** 10.1186/s13075-017-1347-4

**Published:** 2017-06-06

**Authors:** Janet E. Pope

**Affiliations:** Department of Medicine, Division of Rheumatology, University of Western Ontario, St. Joseph’s Health Care, 268 Grosvenor Street, London, ON N6A 4V2 Canada

**Keywords:** Systemic sclerosis, Pulmonary arterial hypertension, Screening, Echocardiogram, Right heart catheterization, Pulmonary function tests

## Abstract

One in six patients with systemic sclerosis will develop pulmonary arterial hypertension (PAH). Screening with echocardiography and possibly pulmonary function testing (to determine the diffusing capacity of carbon monoxide) is recommended to detect PAH at a less severe stage. However, real-world screening programs have problems. Registries where echocardiograms are to be performed annually should have the best-case scenario of nearly perfect screening and referral for right heart catheterization of those highly suspect for PAH. However, registries demonstrate care gaps where patients are not referred for appropriate confirmatory testing when PAH is suspected.

## Introduction

Fifteen percent of systemic sclerosis (SSc) patients develop pulmonary arterial hypertension (PAH) which is fatal [1, 2]. Regular screening is usually performed by echocardiography with estimation of the pulmonary artery pressure [3]. The right heart size and contractility are reported which may occur with PAH, and the presence of pericardial effusions are more frequent in SSc patients with PAH [3]. Screening SSc patients with serial echocardiograms allows for earlier detection of PAH [4]. PAH diagnosis in SSc is often preceded by an inappropriately low diffusing capacity of the lung for carbon monoxide (DLCO), and a ratio of % predicted forced vital capacity (FVC) to % predicted DLCO > 2 should raise suspicion of PAH in SSc [5].

Morrisroe et al. utilized a multi-site SSc longitudinal study of 1636 SSc patients from the Australian Scleroderma Cohort Study, where 12% had PAH proven by right heart catheterization [6]. More than 80% of patients with PAH were initially detected by screening. The annual incidence of PAH was 0.9%. They found that annual PAH screening using the Australian ECHO-based algorithm was less than 50% accurate. Interestingly, patients within this cohort are to be screened annually for PAH including echocardiography and pulmonary function testing (to screen for a low DLCO). This is likely the best-case scenario for screening because procedures are mandated annually in the registry. Low screening rates for PAH in SSc patients are comparable with the Canadian Scleroderma Research Group database (CSRG); where an echocardiogram was ever performed in around 90% of patients whereas follow-up annual echocardiograms were done in 60% of patients over time [7].

Screening is important because there are no reliable risk factors for SSc. Age is a likely risk (older age of onset and/or longer disease duration). Low diffusion capacity is associated with current and/or future PAH; however, this is likely not a risk factor but a finding in PAH that decreases before clinically relevant PAH. Several other reported associations of PAH are not consistent such as anti-centromere antibody, telangiectasia, interstitial lung disease (which can cause PAH from hypoxia), and digital ulcers (another vascular bed that has damage) [5, 8].

### How should you screen?

There are multiple screening algorithms in SSc which may include annual echocardiograms and pulmonary function tests [3, 6, 7]. An algorithm has been developed for prevalent SSc patients who have a low DLCO to determine who should receive an echocardiogram [8]. Many SSc clinicians perform serial echocardiograms every one to a few years (with or without pulmonary function tests to determine whether the DLCO is low as a low DLCO increases the probability of pulmonary hypertension (PH), but is not specific for PH and can be reduced in interstitial lung disease [7].

### Why screen if a patient has no symptoms?

Currently there are no approved treatments for asymptomatic PAH. Therefore, if a patient with SSc truly has no symptoms such as dyspnea, screening could be irrelevant. However, several SSc patients have accommodated to a very sedentary lifestyle due to involvement of multiple other systems. Therefore, they may not complain of dyspnea. Also, even if someone is very active and has no symptoms, results of an echocardiogram can help to predict future development of symptomatic PAH. This needs to be balanced by the probability of false positive screening tests which may occur [3].

### Is pulmonary hypertension more common in limited or diffuse subsets?

In this study, the prevalence of PAH was the same in both the lcSSc and the dcSSc subsets [6]. PAH occurs with long disease duration (so survivors are often those with lcSSc), older age of onset (which is more common in lcSSc than dcSSc where the latter onset on average 10 years younger), and telangiectasia, and anti-centromere may be associated with PAH [5, 8, 9]. PH classification has several groups [10]. Group 1 includes PAH that has several causes including association with connective tissue disease. Group 3 is PH due to lung diseases and/or hypoxia. PH may be part of Group 1 (PAH) in either SSc subset, but PH in Group 3 (lung disease/hypoxia) is more common in dcSSc because this subset has more ILD and more severe ILD than lcSSc [5].

### How is PAH in SSc treated?

Untreated, PAH may be rapidly lethal in SSc [2, 9]. Those patients with a very low diffusing capacity (impaired gas exchange) have higher mortality [9]. There are treatment guidelines for patients who have symptoms (Functional Class II, III, IV) [11]. Trends are for regular screening, early diagnosis by right heart catheterization, and targeted therapy often adding drugs to improve symptoms, signs (dyspnea, heart failure, walk distance), and survival.

## Conclusion

Screening for PAH in SSc allows for earlier detection and treatment that prolongs survival and improves symptoms but it is important that clinicians who follow SSc patients screen and act upon the results, such as referring suspected PAH for right heart catheterization and treatment at an expert center.

## Abbreviations

CSRG: Canadian Scleroderma Research Group
dcSSc: Diffuse cutaneous systemic sclerosis
DLCO: Diffusing capacity of the lung for carbon monoxide
lcSSc: Limited cutaneous systemic sclerosis
PAH: Pulmonary arterial hypertension
SSc: Systemic sclerosis
